# Labeling glycans on living cells by a chemoenzymatic glycoengineering approach

**DOI:** 10.1242/bio.021600

**Published:** 2017-05-12

**Authors:** Ruben T. Almaraz, Yanhong Li

**Affiliations:** Department of Chemistry, University of California, Davis, One Shields Avenue, Davis, CA 95616, USA

**Keywords:** Glycosyltransferases, Glycobiology, Chemoenzymatic, Glycoengineering

## Abstract

Structural glycobiology has traditionally been a challenging field due to a limited set of tools available to investigate the diverse and complex glycan molecules. However, we cannot ignore that glycans play critical roles in health as well as in disease, and are present in more than 50% of all proteins and on over 80% of all surface proteins. Chemoenzymatic glycoengineering (CGE) methods are a powerful set of tools to synthesize complex glycans, but the full potential of these methods have not been explored in cell biology yet. Herein, we report the labeling of live Chinese hamster ovary (CHO) cells by employing three highly specific glycosyltransferases: a sialyltransferase, a galactosyltransferase, and an *N*-acetyl-glucosaminyl transferase. We verified our results by bio-orthogonal blots and further rationalized them by computational modeling. We expect CGE applications in cell biology to rise and their implementation will assist in structural-functional discoveries in glycobiology. This research will contribute to this effort.

## Abstract (precis)

**Summary:** A novel chemoenzymatic glycoengineering method was developed to selectively modify and label surface glycoconjugates in living cells by implementing glycosyltransferases and azido-modified activated sugar analogs.

## INTRODUCTION

The glycobiology of any given cell plays essential roles in many aspects of the cell daily functions. In recent years, we have identified some of those functions as a result of novel innovations in our ability to modify and visualize glycoconjugates. For example, in the area of cancer biology, antibodies such as the MECA-79 (Pablos et al., 2005), HECA-452 (Toppila et al., 1999), and lectins such as the MAA, SNA, and PHA-L (Belardi and Bertozzi, 2015) have identified motifs of vital importance in the detection and progression-tracking of the disease. Metabolic glycoengineering (MGE), which consists in the ability to implement non-natural monosaccharides analogs, was introduced in 1992 and since then a plethora of publications utilizing MGE technology has emerged (Nischan and Kohler, 2016). More recently, chemoenzymatic glycoengineering (CGE) methods became an important part of the toolbox to visualize and consequently study the glycome (Mbua et al., 2013; Zheng et al., 2011). These methods utilize enzymes, especially glycosyltransferases (GTs), to catalyze the transfer of a sugar residue from its activated sugar nucleotide donor to a variety of acceptor molecules. CGE complements MGE and has the added potential of being highly specific, controlling the acceptor, donor sugar, and glycosylic linkage. Nature uses an array of different GTs to create and to constantly and specifically change the composition of its diverse display of surface glycans on the surface of the cell. In the laboratory, GTs have been intensively implemented in the synthesis of complex carbohydrates (McArthur and Chen, 2016) and in the glycosylation of small natural molecules (Bowles et al., 2005; Thuan and Sohng, 2013). However, the use of these novel methods to engineer glycans in living cells has been limited. To our knowledge, only two studies have implemented CGE to modify surface cell glycans in living cells. Zheng et al. (2011) employed a recombinant *Helicobacter pylori* 26695 β1–3–fucosyltransferase to label the *N*-acetyllactosamine (LacNAc) epitopes in a Hank's buffer salt solution (Zheng et al., 2011). More recently Mbua et al. (2013) employed a *Neisseria meningitis* sialyltransferase to sialylate the cell surface glycoconjugates (Mbua et al., 2013). These seminal studies were the origins of CGE by demonstrating the ability to implement GTs *in vitro*. However, more work needs to be done to take advantage of the versatility of GTs and to employ them in more physiological-like conditions for post-modification analysis such as migration and adhesion assays.

In this study, we implemented three unique glycosyltransferases whose functions had been substantiated in effectively and specifically incorporating sialic acid, Gal, and GlcNAc residues in one-pot multi-enzyme reactions. Our first enzyme is the PmST1_M144D, a highly specific α2-3-sialyltransferase from *Pasteurella multocida*, which was employed in the generation of sialyl Lewis X (sLe^X^) structures (Sugiarto et al., 2012). The NmLgtB, a β1-4-galactosyltransferase from *Neisseria meniningitidis*, catalyzes the transfer of Gal residue from the sugar nucleotide UDP-Gal to the acceptor such GlcNAc or Glc-containing glycans (Lau et al., 2010; Li et al., 2016). The NmLgtA, a β1–3-*N*-acetylglucosaminyltransferase from *Neisseria meningitidis*, catalyzes the introduction of GlcNAc from UDP-GlcNAc to acceptor molecules forming β1-3-linkages (Li et al., 2016). The acceptors are the cell surface glycans from several Chinese hamster ovary (CHO) cells. We selected a panel of widely used glycosylated mutant cells that consisted of the CHO K1, Lec2, Lec8 and the Lec12 cell lines (Patnaik and Stanley, 2006). More importantly, the surface *N*- and *O*-glycan structures for the wt CHO, Lec2 and Lec12 had been determined (North et al., 2010), as well as the *N*-glycans structures of Lec8 (Kawar et al., 2005). Their human-like glycobiology (Hossler et al., 2009) made them attractive cell models for the evaluation of live cell labeling by chemoenzymatic glycoengineering; we emphasize, however, that the investigational approach reported in this paper is not limited to CHO cells but can be applied widely to any category of cells.

Here, we specifically aimed to image these different glycobiology mutant CHO cells with the aforementioned glycosyltransferases under physiological-like conditions. The labeling assay was performed in real time with live cells without significant cytotoxicity and it can be further optimized depending on the application.

## RESULTS AND DISCUSSION

**Fig. 1.**
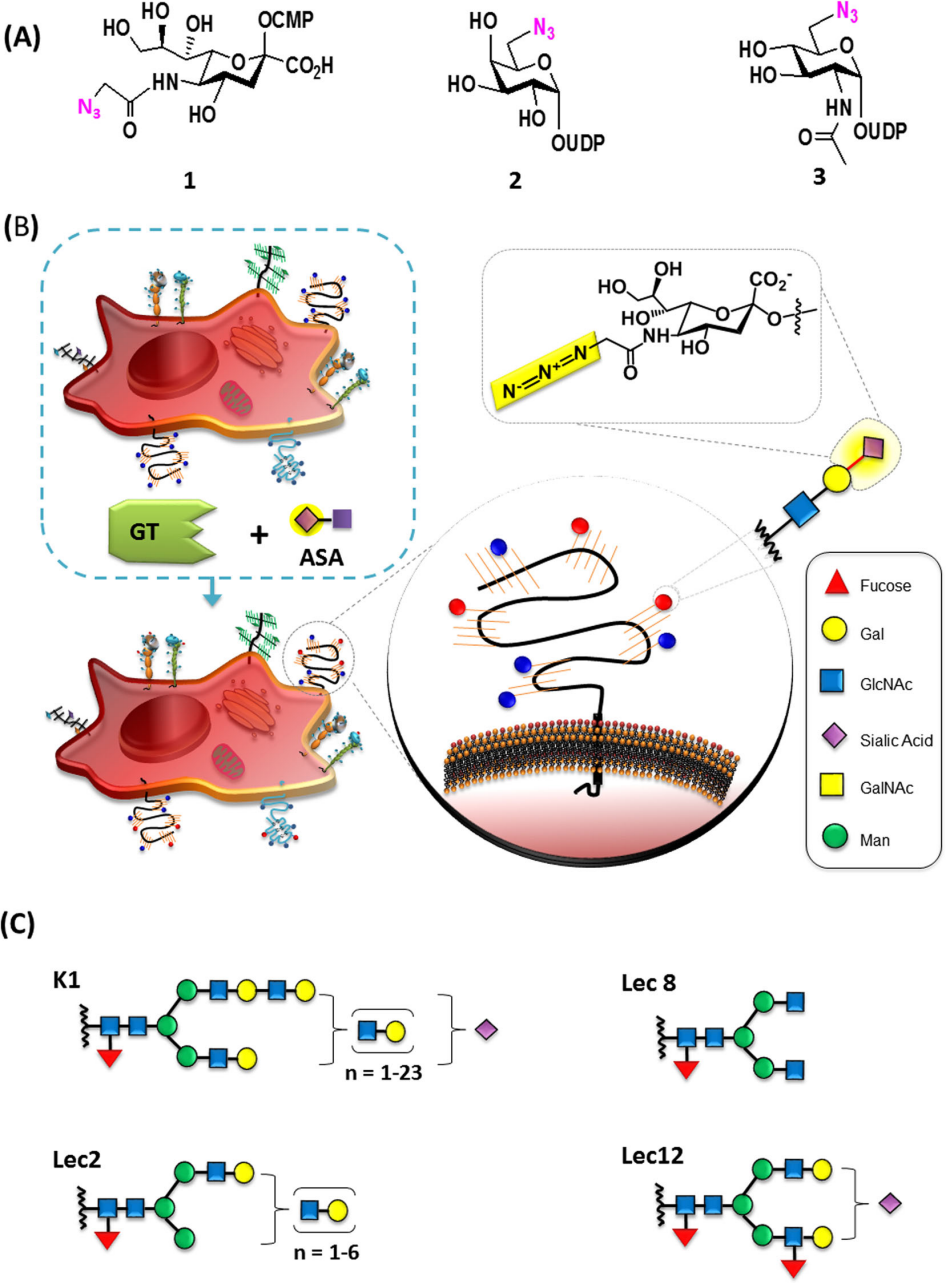
**Labeling surface glycans on living cells.** (A) Sugar nucleotides used in this study. 1, CMP-Neu5Az; 2, UDP-6N_3_Gal; 3, UDP-6N_3_GlcNAc. (B) Illustration of the GCE approach where living cells, in the presence of a glycosyltransferase (GT) with its respective activated sugar analog (ASA), modify cell-surface glycans under cell culturing conditions. Azide analogs are attractive because of their low background, specificity, and the chemistry to conjugate different groups to the azide group is well known. (C) Cartoon interpretations of most likely *N*-glycans structures found on the CHO K1, Lec2, Lec8, and Lec12 cells. Structures were taken from Patnaik and Stanley (2006).

An illustrative diagram of the chemoenzymatic glycoengineering approach is shown in Fig. 1A,B. The glycan structures of the most abundant species in each of our model CHO cells are shown in Fig. 1C. The first enzyme, PmST1_M144D, was expressed and purified as previously described (Sugiarto et al., 2012). We then sought to determine its catalytic activity in cells bearing LacNAc terminal residues such those expressed on the Lec2 cells. We also expected this enzyme to glycosylate, albeit to a lesser extent, the K1 and Lec12 cells, since both cells have traces of terminal LacNAc on their surface glycans (North et al., 2010). Accordingly, we incubated the CHO K1, Lec2, Lec8 and Lec12 cells with PmST_M144D and different concentration of CMP-Neu5Az 1 (Fig. 1A) at 37°C for 10 and 20 min in a DPBS-Tris buffer.

Our findings revealed that PmST1_M144D effectively incorporated the Neu5Ac analog, Neu5Az, onto the surface glycoconjugates bearing LacNAc terminal substrates in living cells and the incorporation of azido modified sialic acid allows for the post modification microscopy analysis (Fig. 1B). The effective glycosylation for the Lec2 cells was found at 500 µM CMP-Neu5Az, 20 min incubation, in the presence of 1 mg/ml PmST1_M144D in a DPBS-Tris matrix. No florescent was detected on our control cells (no enzyme present). These conditions were demonstrated to be safe for the cells since we noted that there were no significant changes in cell growth and morphology (Fig. S3). All cells were visualized under similar technical conditions and the analysis was done relative to the Lec2 cells. Lower donor concentrations, 100 µM CMP-Neu5Az, were tested and limited fluorescence was noticed in the Lec2 cells, and none on the K1, Lec8, and Lec12 cells. Similar data was observed at 10 min incubation periods. At optimal Lec2 labeling conditions, we detected limited fluorescence on the CHO K1 and none on the Lec8 nor on the Lec12 cells. It was interesting to see labeling differences between the K1 and Lec12 cells, because on a previous study, the PmST1_M144D sialylated both LacNAc and fucosylated LacNAc structures efficiently, the major difference between these two cell lines (Sugiarto et al., 2012). It is noteworthy that we detected fluorescence when Lec12 cells were incubated for 30 min. However, it is unknown if it sialylated LacNAc or fucosylated LacNAc or both structures since both types can be found on the Lec12 cells (North et al., 2010). To validate our CGE methodology, we confirmed the incorporation of non-natural sugars implementing GTs using bio-orthogonal glyco blots (Fig. S4). The blots eliminated the possibility of non-specific binding of the fluorophore to the cell membrane. It also eliminated the possibility of indigenous GTs acting on our non-natural activated sugar donors under our assay conditions.

**Fig. 2.**
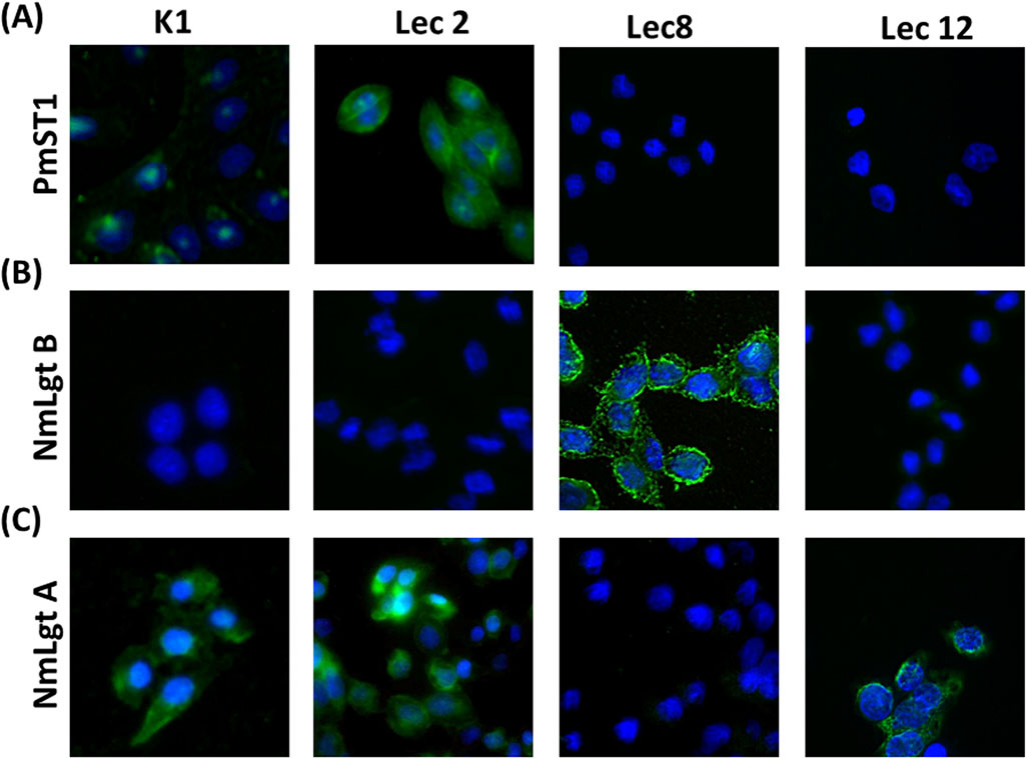
**Glycobiology mutant CHO cells labeled by chemoenzymatic glycoengineering.** K1, Lec2, Lec8 and Lec12 CHO cells incubated for 20 min at 37°C in the presence of (A) the mutant *P.* *multocida* sialyltransferase (PmST1_M144D)+CMP-Neu5Az, (B) *N. meningitidis* β1,4-galactosyltransferase (NmLgtB)+UDP-6N_3_Gal, and (C) *N. meningitidis* β1–3-*N*-acetylglucosaminyltransferase (NmLgtA)+UDP-6N_3_GlcNAc. After enzymatic treatment, cells were then washed, fixed and click-chemistry reacted with biotin alkyne. Cells were then visualized with streptavidin-FTCI on a Zeiss Eclipse microscope.

After successfully sialylating LacNAc-bearing cells, we proceeded in extending our approach implementing a galactosyltransferase. The galactosyltransferase NmLgtB was expressed and purified as described (Lau et al., 2010). We incubated NmLgtB in the presence of UDP-6N_3_Gal (Fig. 1A) for 20 min at 37°C. Then, by conjugating biotin-alkyne via click-chemistry, we were able to demonstrate the incorporation of 6N_3_Gal on the surface glycans on the Lec8 cells (Fig. 2B). We detected low to no yields of fluorescent on the Lec2, Lec12 cells and K1 cells under similar visualization conditions. Having effectively incorporated Gal residues into the Lec8 cells, we next examined the effect of incubating the same set of cells with the β1-3-*N*-acetylglucosaminyl transferases. For this purpose, the NmLgtA was expressed, purified (Blixt et al., 1999) and incubated with the CHO cells in the present of UDP-6N_3_GlcNAc. Our analysis revealed that NmLgtA incorporated the azido-modified GlcNAc sugar on our set of CHO cell-surface glycans with the exception of the Lec8 cell line (Fig. 2C). This was not unexpected because the Lec8 cell-surface glycans are composed of the core octose (Fig. 1C), not suitable ligands for NmLgtA.

**Fig. 3.**
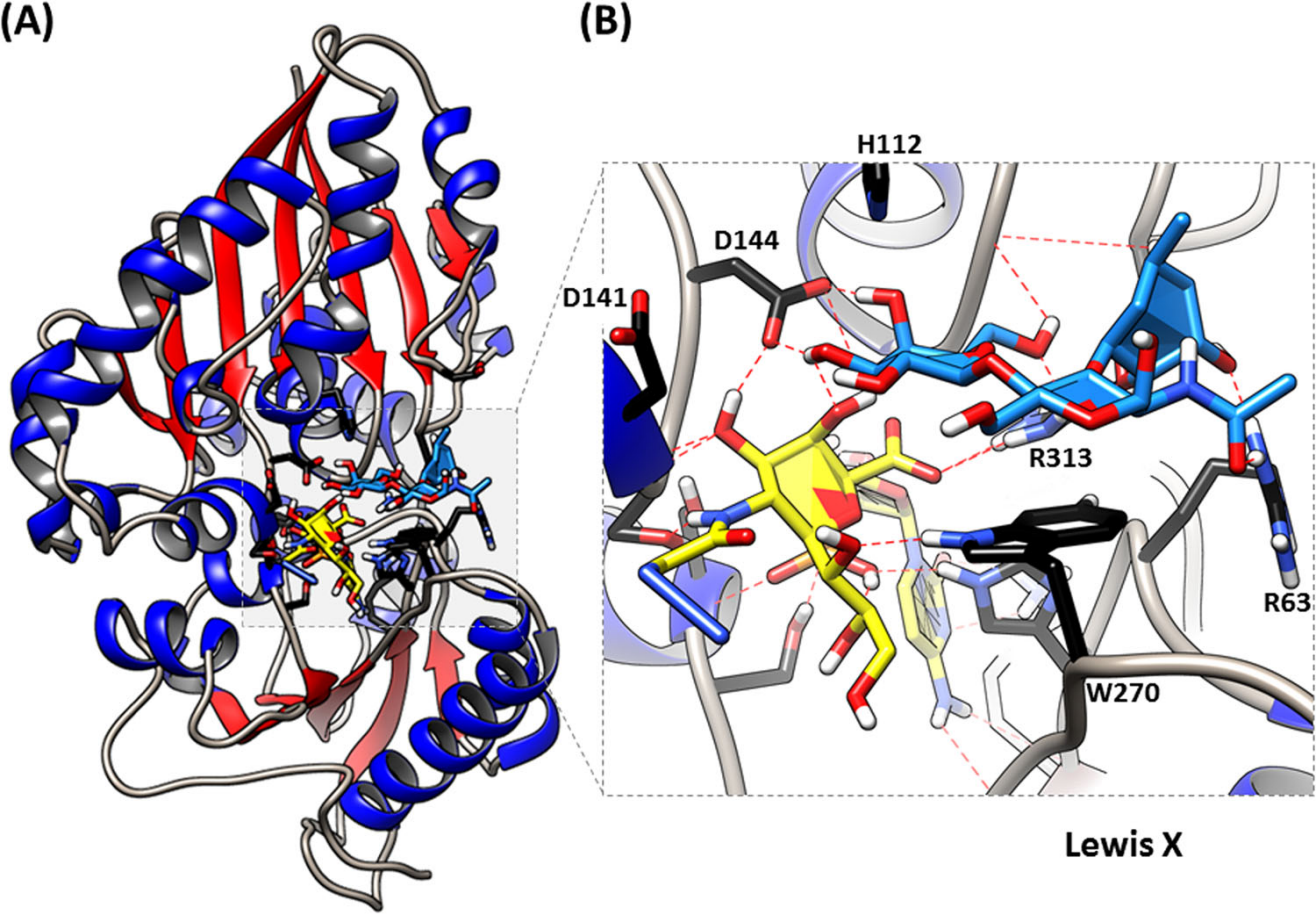
**Structural analysis of PmST1_M144D.** (A) The complex structure of the PmST1 bound to CMP-Neu5Az (yellow) and Lewis X (dodger blue). (B) Closer view of the catalytic site bound to donor CMP-Neu5Az and sugar acceptor, Lewis X. Amino acids interacting with the sugar are shown in black sticks. Beta sheets are shown in red and helices in blue. Hydrogen bonds are shown in red dotted lines. Mutation and energy minimization was done with Scigress FJ 2.6. Figures were generated by UCSF Chimera 1.11.

The three glycosyltransferases successfully incorporated a synthetic sugar on the selective CHO cells under cell culturing-like condition in 20 min. However, during the sialylation by PmST1_M144D, there was a significant difference between K1 and Lec12 labeling and it was unclear if this variance was due to the length of the glycan chain or the presence of fucose. To shine light on this issue, we examined the Asp–Met substitution within PmST1_M144D and how this affected the sialylation of K1 and Lec12 cells, by creating a model from the complex PmST1-CMP-Nau5Ac-lactose crystal structure in which the amino acid at position 144 was mutated to aspartic acid. The acceptor sugar was modified to LacNAc and then to Le^X^. Our model demonstrated favorable interactions with LacNAc as hydrogen bonds formed between its *N*-acetyl amino group and R313, demonstrating a preference for LacNAc over lactose (Fig. 3A,B). The model shows the D144 residue is in close proximity to the catalytic groups which explains the effect on the PmST1 reduced sialidase activity as previously discussed (Sugiarto et al., 2012). The catalytic site chemistry of PmST1 involves the formation of a glycosidic linkage between the second hydroxyl group on Neu5Ac/z of the donor (CMP-Neu5Az) and carbon C2 of the acceptor Gal residue, both in close proximity to D141 and now in a more electronegative environment due to the M144D mutation. However, fucose seems unaffected by the presence of D144. In fact, it is a monosaccharide away from either D144 or D141. The model complex structure does, however, demonstrate the feasibility to accommodate fucose into the binding site and the creation of addition hydrogen bonds with R313 and R63. The methyl group of the fucose favorably interacts with the backbone of P34, L82, K83, and D84. Thus, it is unlikely that fucose inhibits sialidase activity by PmST1_M144D or by PmST1. On the other hand, the structural differences between the surface K1 and Lec12 surface glycans are significant (Fig. 1C). Moreover, based on reported MALDI-MS data, on wild-type CHO cells and Lec12 cells, their *N*-glycans do not only differ on the Lec12 cell overexpression of fucosylated structures but on the wild-type CHO having longer glycans, bearing up to 26 LacNAc units. Additionally, there was a 0.03 abundance of the bisecting GlcNAc on the Lec12 but no trace of it was found on the wild-type CHO cells. Therefore, it is reasonable to speculate that the structure combination of fucosylation, short glycans, and traces of bisecting GlcNAc, delays interactions between the ligand right conformation(s) and the sialyltransferase catalytic site. Hence, the ability of sialylating Lec12 was only detected when incubating for 30 min with PmST1_M144D.

In conclusion, we described a novel approach of labeling glycans by chemoenzymatic incorporation of non-natural sugars on living cells. Taking together, this study showed that glycosyltransferases, previously characterized in one-pot multi-enzyme reactions, can also glycosylate living cells. Notably, the glycoenzymatic technique offers an alternative method for labeling live cell surface glycans with more specificity, enhancing our ability to visualize the glycome. Also, one can envision adaptations of the method to incorporate unique structures (e.g. sLe^X^) to explore their biological relevance. We predict that these techniques will greatly aid in the interrogation of glycoprotein function in living systems.

## MATERIALS AND METHODS

### Materials

All reagents were from Sigma-Aldrich (St. Louis, MO, USA) unless otherwise stated. The synthesis of the activated sugar analogs, CMP-Neu5AcN_3_, and UDP-6N_3_GlcNAc used in this study have been reported elsewhere (Yu et al., 2004). Their identity and purity were verified for this project (Figs S1 and S2). The synthesis of UDP-6N_3_Gal is currently in preparation for publication (H. Yu unpublished data). Alkyne biotin was purchased from Click Chemistry Tools LLC (Scottsdale, AZ, USA). Streptavidin-fluorescein isothiocyanate (FITC) was purchased from Vector Laboratories Inc. (Burlingame, CA, USA). The mutant of *Pasteurella multocida* sialyltransferase (PmST1_M144D), *Neisseria meniningitidis* β1–4-galactosyltransferase (NmLgtB), and *N. meningitidis* β1–3-*N*-acetylglucosaminyltransferase (NmLgtA) were obtained as previously described (Lau et al., 2010; Li et al., 2016; Sugiarto et al., 2012; Yu et al., 2005).

### Tissue culture/cell growth conditions

The CHO Lec2, Lec8, and Lec12 cells were kindly supplied by Professor Pamela Stanley (Albert Einstein College of Medicine, USA). These cells were grown in monolayer in alpha MEM medium (GIBCO 11900-073) supplemented with 10% Fetal Calf Serum (Sigma-Aldrich) and 1% (v/v) Penicillin and Streptomycin (Invitrogen). The CHO K1 cells were kindly provided by Professor Jon Sack (University of California Davis, USA) and cultured in F-12K Medium (GIBCO 21127022) supplemented with 10% Fetal Bovine Serum (GIBCO 16000044) and 1% (v/v) Penicillin and Streptomycin (Invitrogen). TrypLE™ Express, and stable trypsin replacement enzyme were purchased from Invitrogen (Carlsbad, CA, USA). In all cases, cells were incubated in a 5.0% carbon dioxide, water saturated incubator at 37°C.

### Cell chemoenzymatic labeling

The enzymes were purified and concentrated in Tris–HCl buffer (100 mM, pH 8.8) containing 20 mM MgCl_2_ and 0.15 M NaCl. Before reactions, each enzyme and substrate were mixed (PmsT1_M144D+CMP-Neu5Az or NmLgtB+UDP-6N_3_Gal or NmLgtA+UDP-6N_3_GlcNAc) in the Tris buffer. We opted not to use Hank's buffer since some of these enzymes (e.g. NmLgtB) have been shown to accept the free GlcNAc and Glc as acceptor, the latter a component of Hank's buffer. The day before the experiments CHO cells were seeded in12-well plates at 37°C and 5% CO_2_ in their respective growth medium. From the enzyme plus substrate solution, 100 µl was added to each well containing the mutant CHO cells. To each well 100 µl of DPBS (Gibco 14040182) was added to give a final volume of 200 µl and enzyme and substrate concentration of 1 mg/ml and 500 µM, respectively, and incubated for 20 min at 37°C. Fresh Click-iT reaction cocktail was prepared for each experiment and each experiment was repeated at least three times. To make a ml of Click reaction mixture, the following was mixed in the same order; 810 µl of Click-iT buffer reaction and 100 µl of Click-iT additive C (Invitrogen), 50 µl of a 50 mM CuSO_4_ solution, 30 µl of a 5 M NaCl, and just prior the labeling 10 µl of a 100 mM biotin Alkyne (Click Chemistry Tools LLC).

Prior to click labeling, the cells were washed with DPBS and fixed with 3.7% formaldehyde for 5 min at room temperature. Cell surface glycan labeling of azido groups was done by adding 200 µl of a freshly prepared Click-iT Reaction Mixture containing 1.0 mM biotin-alkyne and incubated at RT for 25 min. After incubation with these reagents, the cells were rinsed three times with PBS containing 5.0% BSA. Then, 200 µl of PBS containing FITC-Streptavidin (Invitrogen) was added to each well and incubated for 45 min at room temperature. Also, 10 µl of a 1.0 mM 4,6-diamidino-2-phenylindole (DAPI) solution was added to each sample to stain the nuclei. The cells were then washed three more times with PBS containing 5.0% BSA. Images were taken using a Nikon eclipse microscope with a 20 and 40× objective (Nikon Inc., Melville, NY, USA). Fluorescence pictures of FITC- and DAPI-labeled cells were recorded for the same exposure time and overly with the Nikon Imaging System. For bio-orthogonal glyco blot analysis, the azido-modified glycoconjugates obtained from chemoenzymatically treated CHO cell were rinsed with DPBS and detached with dissociation buffer and transferred to 1.5 mL Eppendorf tubes and washed twice with 1.0 ml DPBS. The cells were then resuspended in 200 µl of Click-iT reaction mixture containing 1.0 mM biotin-alkyne (Click Chemistry Tools LLC) and incubated for 25 min at room temperature. Cells were then washed, lysed for SDS-PAGE and blotted as previously reported (Almaraz et al., 2012).

### General computational modeling approaches

The availability of the PmST1 crystal structure prompted us to examine the M144D mutation effect on the binding and sialylation mechanism of fucosylated versus unfucosylated LacNAc structures (Fig. S5). Computational modeling was done with the SCIGRESS version FJ 2.6 software package (Fujitsu Ltd., Tokyo, Japan). All computations were performed on a Cyber power computer equipped with an Intel^®^ Core™ i7-3970X Extreme Edition Six-Core 3.50 GHz, >20% overclock processor, and 64GB of RAM (Cyber Power PC Inc, CA, USA). The 3D structure of the PmTS1 3D was taken from the protein databank (PDB ID: 2IHZ). The starting glycan structure was lactose to which *N*-acetyl amino group and a fucose residue were added to the second and third hydroxyl group respectively on the glucose residue. The complex was first energy optimized with the MM3 force field and this complex was used for MD structure optimization.
